# Peripheral adaptive immune cell counts and mortality in adult sepsis: a systematic review and meta-analysis

**DOI:** 10.3389/fimmu.2026.1937759

**Published:** 2026-09-01

**Authors:** Yiyuanzi Zhao, Xue Gong, Wei Bi, Jiaqi Wang, Caijun Wu

**Affiliations:** 1 Department of Emergency Medicine, Dongzhimen Hospital, Beijing University of Chinese Medicine, Beijing, China; 2 Institute of Sepsis, Beijing University of Chinese Medicine, Beijing, China

**Keywords:** absolute lymphocyte count, CD4, CD8, meta-analysis, mortality, sepsis, systematic review, T lymphocytes

## Abstract

**Background:**

Lower peripheral lymphocyte counts are associated with mortality in sepsis, but evidence across related adaptive immune-cell compartments has not been systematically quantified.

**Methods:**

Nine databases were searched from inception through 7 August 2026 using expanded controlled-vocabulary and free-text terms for sepsis, lymphocyte/T-cell subsets, absolute cell counts, and mortality. Observational studies comparing absolute peripheral CD4+ T-cell, CD8+ T-cell, CD3+ T-cell, or absolute lymphocyte counts (ALCs) between adult non-survivors and survivors were included. Random-effects mean differences (MDs) were estimated using restricted maximum likelihood with a modified Hartung-Knapp adjustment. Prediction intervals, restricted and leave-one-out analyses, ratio-of-means analyses, Egger tests, and exploratory meta-regression were also performed. Results: Thirteen studies were included. Counts were lower in non-survivors for CD4+ T cells (13 studies; MD = -86.06 cells/µL, 95% CI -127.29 to -44.83; I² = 77.0%), CD8+ T cells (11 studies; MD = -41.83, 95% CI -68.37 to -15.29; I² = 61.9%), CD3+ T cells (10 studies; MD = -133.99, 95% CI -220.41 to -47.56; I² = 88.3%), and ALCs (10 studies; MD = -201.46, 95% CI -323.04 to -79.88; I² = 71.7%). All prediction intervals crossed zero. CD4 remained lower after exclusion of studies classified as high risk in the descriptive QUIPS synthesis; corresponding CD8, CD3, and ALC estimates remained negative but were imprecise because only four, two, and three lower-risk studies remained. After excluding statistically converted data, CD8 was no longer significant and ALC evidence was based on only two studies. Adjusted analyses varied across studies and did not consistently support independent prognostic associations. Evidence robustness was greatest for CD4; CD8, CD3, and ALC were more sensitive to data conversion, heterogeneity, or sparse lower-risk evidence.

**Conclusion:**

Adult sepsis non-survivors had lower absolute counts across lymphocyte and T-cell compartments. The findings are most consistent for CD4, whereas CD8 and ALC estimates are more dependent on converted data. Heterogeneity, geographic concentration, and prediction intervals crossing zero limit direct clinical generalization and support the need for standardized longitudinal studies with severity-adjusted analyses.

**Systematic Review Registration:**

https://www.crd.york.ac.uk/PROSPERO/, identifier CRD420261446443.

## 1 Introduction

The threat posed by sepsis extends beyond the invading infection to the dysregulated host response it provokes. According to Sepsis-3, sepsis is life-threatening organ dysfunction caused by a dysregulated host response to infection (1). In 2017, an estimated 48.9 million cases of sepsis occurred worldwide and were associated with approximately 11.0 million deaths, accounting for nearly one-fifth of all global deaths (2). Sepsis, however, does not follow a single pathophysiological trajectory; it is a highly heterogeneous syndrome shaped by the anatomical source of infection, causative pathogens, baseline host characteristics, and the evolving immune response. Identifying measurable host-response phenotypes associated with organ dysfunction and death remains central to improving risk stratification and advancing precision treatment.

Contemporary immunology has moved beyond the simplified biphasic model of “early hyperinflammation followed by late immunosuppression.” Hyperinflammation, immune tolerance, defective antigen presentation, metabolic reprogramming, and lymphocyte death can coexist from the early stages of sepsis and evolve with the infection source, host condition, and treatment course (3, 4). Tissue studies in patients who died from sepsis have demonstrated marked loss and functional impairment of adaptive immune cells in the spleen and lung (5), while earlier human studies showed progressive and profound depletion of B cells and CD4+ T cells (6). Adaptive immune impairment may therefore be a core pathological process that develops alongside persistent infection, secondary infection, organ dysfunction, and adverse outcomes, rather than a secondary event that appears only after inflammation resolves.

Peripheral blood lymphopenia is among the most clinically accessible manifestations of this immune disturbance. Persistent lymphopenia has been associated with increased mortality in sepsis (7), but the absolute lymphocyte count (ALC) represents a broad lymphocyte pool and cannot distinguish the relative contributions of T cells, B cells, and natural killer cells. Absolute CD3+, CD4+, and CD8+ T-cell counts provide progressively greater resolution of the total T-cell, helper T-cell, and cytotoxic T-cell compartments. Most available clinical studies are single-center observational cohorts and differ in the reported cell populations, sampling times, outcome definitions, and data formats. It therefore remains uncertain whether adverse-outcome-associated cell loss is confined to a single T-cell subset or reflects a broader contraction of adaptive immune cell pools, and which marker provides the most consistent evidence.

Blood transcriptomic and single-cell studies further indicate that similar cell counts can conceal biologically distinct immune states. Patients with sepsis can be classified into molecular endotypes with different levels of adaptive immune activity and different mortality risks (8, 9), while single-cell transcriptomics has uncovered variation in cellular composition and state that is not captured by bulk blood measurements (10). A recent single-cell multi-omic study of 281 adults and children demonstrated infection-source- and age-specific immune features in sepsis: an NR4A2+ central memory CD4+ T-cell subset was associated with poorer survival, whereas proinflammatory CD8+ T-cell subsets expressing CCL3, CCL4, and TNF expanded in selected infection sources (11). Thus, contraction of the overall cell pool can coexist with selective expansion or dysfunction of specific subsets. Likewise, an increased proportion of regulatory T cells (Tregs) within the CD4+ compartment may coexist with a lower absolute Treg count, highlighting that cell proportions, absolute numbers, and functional state are not interchangeable (12).

Although high-dimensional omics substantially improves the resolution of immune phenotyping, cost, technical complexity, and turnaround time limit routine clinical use. In contrast, ALC and absolute CD3, CD4, and CD8 counts are widely accessible and may provide a first-line bridge between bedside assessment and deeper immune profiling. Precision immunotherapy in sepsis similarly requires reproducible host-response markers for prognostic and predictive enrichment rather than indiscriminate immune stimulation (13, 14). Recombinant human interleukin-7 increases circulating lymphocyte counts in septic shock with lymphopenia, providing proof of concept that adaptive immune cell reserve may be therapeutically modifiable, although clinical benefit remains uncertain (15).

We therefore conducted a systematic review and meta-analysis of the association between peripheral blood absolute ALC, CD3+ T-cell, CD4+ T-cell, and CD8+ T-cell counts and mortality in adults with sepsis or septic shock. CD4 served as the primary count-based marker because it was reported in all 13 included studies; CD8, CD3, and ALC were treated as secondary or supporting analyses. We quantified absolute and relative between-group differences, calculated prediction intervals, examined sensitivity to data transformation, sampling time, language, special populations, ambiguous dispersion reporting, and potential cohort overlap, and summarized study-level adjusted evidence. The aim was to characterize the strength and limitations of these count-based associations while distinguishing numerical cell loss from functional immune states.

## 2 Methods

### 2.1 Study design and registration

This systematic review and meta-analysis was designed, conducted, and reported in accordance with the PRISMA 2020 statement (16). The protocol was registered in PROSPERO (CRD420261446443). The PICOS framework comprised adults aged 18 years or older with sepsis or septic shock; peripheral blood absolute CD4+ T-cell counts as the primary exposure, with CD8+ T-cell, CD3+ T-cell, and ALC as secondary/supporting exposures; survivors as the comparator; 28-day, 30-day, in-hospital, or intensive care unit mortality as outcomes; and observational clinical studies reporting extractable grouped continuous data as eligible designs. Because all included studies reported mortality, the manuscript terminology was standardized to mortality rather than the broader term adverse outcomes.

### 2.2 Information sources and search strategy

PubMed, EMBASE, Web of Science Core Collection, the Cochrane Library, China National Knowledge Infrastructure (CNKI), Wanfang Data, VIP, SinoMed, and the Chinese Medical Journal Full-text Database were searched from inception through 7 August 2026. The final strategies combined controlled vocabulary and free-text terms for sepsis or septic shock, CD3/CD4/CD8 and lymphocyte/T-cell subsets, absolute lymphocyte or cell counts, and mortality, survival, prognosis, or prediction. Equivalent Chinese terms were used in the Chinese databases. No language or publication-year restriction was applied. Search syntax was adapted to each database and the complete strategies and retrieval counts are reported in **Supplementary Table 1**. Conference abstracts and registry records without published grouped absolute-count data were not included in the quantitative synthesis.

### 2.3 Eligibility criteria

Studies were eligible if they (1): enrolled adults with sepsis or septic shock (2); reported absolute CD4+ and/or CD8+ T-cell counts, with or without absolute CD3+ T-cell counts or ALC (3); provided data stratified by mortality versus survival (4); reported means and standard deviations, standard errors, medians and interquartile ranges, or other continuous data that could be converted to means and standard deviations; and (5) used a prospective or retrospective observational clinical design.

Studies were excluded if they (1): enrolled children, neonates, or non-adult populations (2); were animal studies, *in vitro* studies, case reports, conference abstracts, reviews, commentaries, or guidelines (3); reported only percentages, ratios, receiver operating characteristic curves, odds ratios, hazard ratios, correlations, or prediction-model outputs without recoverable grouped absolute counts (4); did not report an eligible outcome grouping (5); focused on special immune states such as HIV infection, organ transplantation, cancer chemotherapy, or long-term immunosuppressive therapy without separately extractable data for general sepsis populations; or (6) represented duplicate reports or records.

### 2.4 Study selection and data extraction

Search records were imported into reference-management software and deduplicated. Two reviewers independently screened titles and abstracts, assessed full texts, and extracted data. Disagreements were resolved through discussion and, when necessary, adjudication by a third reviewer. Extracted variables included first author, publication year, country or region, participating center, recruitment period, study design, patient setting, sepsis definition, sampling anchor and timing, mortality endpoint, group sample sizes, flow-cytometry platform and counting method, and grouped values for CD4+ T cells, CD8+ T cells, CD3+ T cells, and ALC. We also extracted whether multivariable analysis was performed, the covariates considered, and whether each absolute count remained associated with mortality after adjustment. Potential cohort overlap was assessed using centers, recruitment periods, author groups, registration identifiers, eligibility criteria, and marker/outcome definitions. Full-text exclusion categories are provided in **Supplementary Table 5**.

### 2.5 Data harmonization and transformation

Only peripheral blood absolute counts were included in the primary analyses, and all units were converted to cells/µL. Values reported as 10^9^/L were multiplied by 1,000. Standard deviations were derived from standard errors as SD = SE × $\sqrt{n}$. Medians and quartiles were converted to means and standard deviations using the Luo and Wan methods (17, 18), which assume that the reported quantiles provide a reasonable approximation to the underlying continuous distribution; this assumption may be imperfect for right-skewed cell-count data. The original statistic type and transformation rule were retained in the machine-readable source dataset. In Li et al. (2025), “TLC” was defined by the original authors as T-lymphocyte count and was therefore assigned to the CD3 compartment, not to ALC. In Yang et al. (2023), the dispersion was labelled as SD, but internal reconstruction showed that interpreting the printed values as SE produced test statistics substantially closer to those reported. The primary analysis therefore used SD = SE × √n, with alternative analyses treating the printed values directly as SD and excluding the study.

### 2.6 Risk of bias and evidence interpretation

Two reviewers independently assessed risk of bias using the Quality In Prognosis Studies (QUIPS) tool across study participation, study attrition, prognostic-factor measurement, outcome measurement, study confounding, and statistical analysis/reporting (19). Domain-level judgments were prioritized because QUIPS does not prescribe a numerical summary score; an overall low, moderate, or high label was used only as a descriptive study-level synthesis, with particular weight given to confounding and analysis/reporting. Formal GRADE certainty categories were not assigned because the pooled effects were predominantly unadjusted survivor–non-survivor contrasts and the included studies did not constitute a uniform phase of prognostic-factor investigation with comparable confounding adjustment, both of which are important considerations when judging prognostic-factor evidence (20). Instead, evidence robustness was summarized descriptively using QUIPS, between-study heterogeneity, 95% prediction intervals, sensitivity to statistical conversion and study exclusion, possible small-study effects, geographic concentration, and the consistency of adjusted analyses reported by individual studies.

### 2.7 Statistical analysis

The primary effect measure was the unadjusted mean difference (MD) in absolute cell count between non-survivors and survivors; negative values indicate lower counts in non-survivors. The primary inferential focus was CD4. CD8, CD3, ALC, RoM, Egger, and meta-regression analyses were secondary or supporting. Random-effects models used restricted maximum likelihood (REML) for τ² and Knapp-Hartung small-sample inference with an *ad hoc* safeguard that prevented the variance scale from falling below the conventional random-effects value (scale = max[1, q*]; equivalent to metafor test = “knha”, adhoc = “se”) (21, 22). Two-sided 95% CIs and P values were calculated with k - 1 degrees of freedom. Prediction intervals were calculated for analyses with at least three studies. Heterogeneity was summarized using Q, I², and τ² (23). Because the pooled MDs were unadjusted, they were not interpreted as adjusted prognostic effects.

Sensitivity analyses were restricted to English-language studies, Chinese-language studies, 28-day or 30-day mortality, explicitly documented sampling within 24 h or on day 1, studies not requiring median/interquartile-range conversion, and cohorts not restricted to older or special populations. Additional analyses excluded Li et al. (Aging), treated the Yang 2023 dispersion directly as SD or excluded that study, and excluded potentially overlapping Li (Front Immunol) and Cheng cohorts in mutually exclusive scenarios. Leave-one-out and Baujat diagnostics assessed influential studies. RoM analyses pooled log mean ratios using the same REML-modified-Hartung-Knapp framework and delta-method variances; a no-conversion RoM sensitivity analysis was also performed. Egger regression and univariable meta-regression were exploratory. No multiplicity adjustment was applied, and secondary P values were interpreted descriptively (24). A *post hoc* risk-of-bias sensitivity analysis excluded studies classified as high risk in the descriptive study-level QUIPS synthesis.

Among studies reporting at least three of ALC, CD3, CD4, and CD8, we also summarized whether counts were lower in at least three reported compartments. Because these compartments are correlated and biologically nested, this *post hoc* analysis was used as a descriptive concordance display rather than a separate inferential test.

## 3 Results

### 3.1 Study selection and characteristics

**Figure 1:**
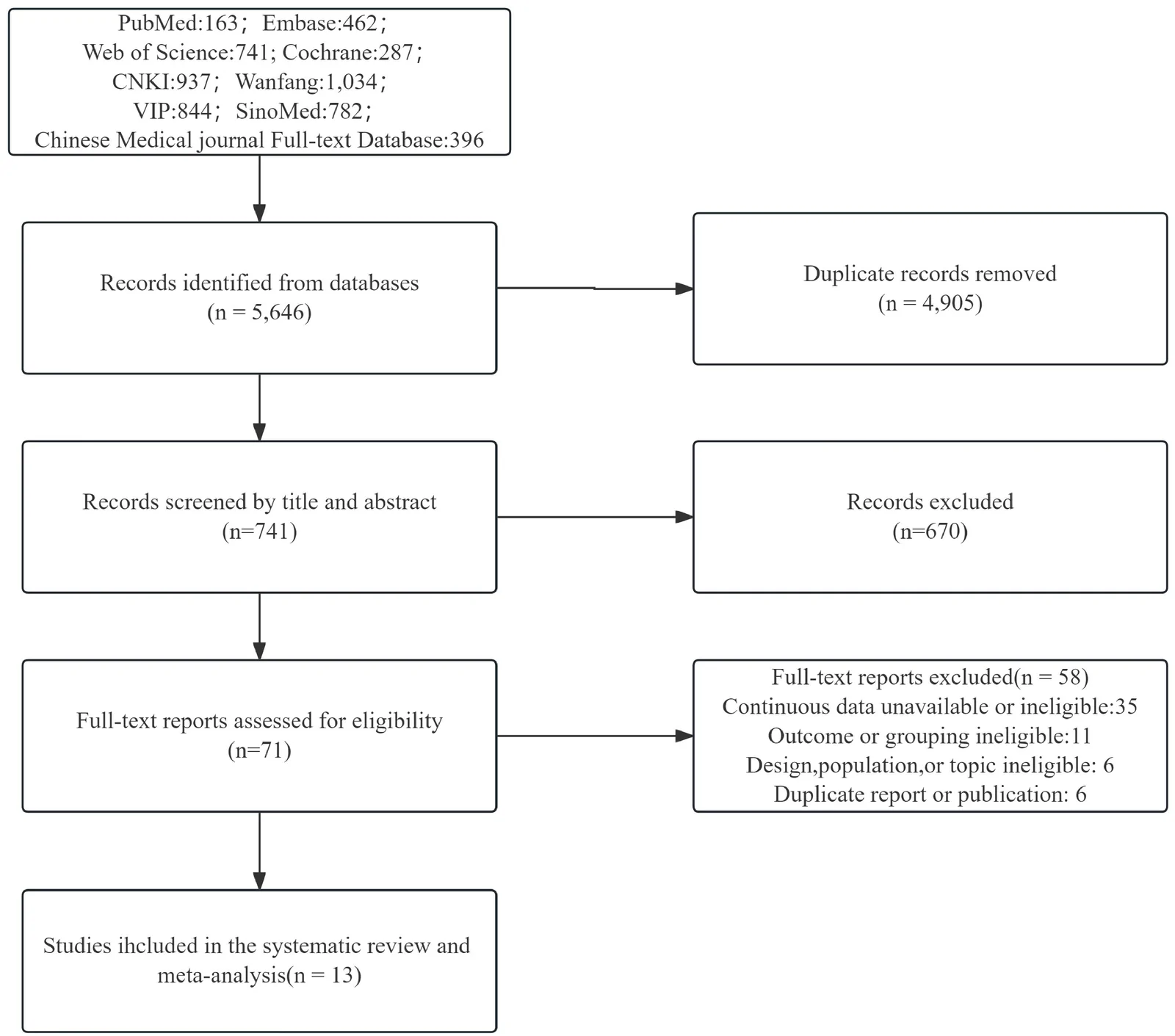
PRISMA 2020 flow diagram. The final expanded searches of nine databases identified 5,646 records; 741 remained after removal of 4,905 duplicates, 71 full-text reports were assessed, 58 were excluded, and 13 mortality studies were included. Full-text exclusion categories are provided in **Supplementary Table 5**.

The database searches identified 5,646 records. After removal of 4,905 duplicates, 741 records underwent title and abstract screening; 71 full-text reports were assessed, 58 were excluded, and 13 studies were included (25–37) (**Figure 1**). Eight studies were published in English and five in Chinese; 11 of 13 cohorts were conducted in China, one in Italy, and one in India. Nine studies assessed 28-day mortality, one assessed 30-day mortality, and three assessed in-hospital mortality. Nine studies explicitly sampled within 24 h or on day 1; four used early but less precisely defined anchors or allowed sampling beyond 24 h. Sepsis definitions included Sepsis-3, earlier consensus criteria, or a Chinese guideline-based definition. Forty-four study-marker records were available: 13 for CD4, 11 for CD8, 10 for CD3, and 10 for ALC. **Table 1** summarizes the primary characteristics; participating centers, recruitment periods, measurement methods, and adjusted evidence are detailed in **Supplementary Tables 2**, **3**.

**Table 1:** Characteristics of the included studies.

Study	Country	Design/setting	Sepsis definition	Sampling anchor	Mortality endpoint	Non-survivor/survivor n	Markers
Polilli 2021	Italy	Retrospective cohort; infectious-disease ward	Sepsis-2/ICD-9-CM-based ascertainment	Hospital entry; exact hours not reported	In-hospital	49/229	ALC, CD3, CD4, CD8
Wei 2021	China	Prospective observational; ICU	Sepsis-3	Day 1 (primary) and day 3	28-day	18/44	ALC, CD3, CD4, CD8
Chen 2022	China	Prospective observational; ICU	Sepsis-3-based ICU cohort	ICU day 1	28-day	13/68	ALC, CD3, CD4, CD8
Yang 2023	China	Retrospective; surgical ICU	Sepsis-3	Morning after SICU admission	28-day	81/198	ALC, CD3, CD4, CD8
Suri 2024	India	Prospective observational; respiratory ICU	Sepsis-3	Enrollment within 48 h of arrival; baseline used	In-hospital	41/59	ALC, CD3, CD4, CD8
Huang 2024	China	Prospective observational; ICU/emergency ICU	Sepsis-3	Within 24 h of enrollment	28-day	29/71	ALC, CD4, CD8
Li (Aging) 2024	China	Retrospective; ICU	Sepsis-3	Admission day 1 (primary) and day 3	28-day	114/342	ALC, CD3, CD4, CD8
Jia 2024	China	Retrospective; emergency department	Sepsis-3	Day 1 or 2 after recognition	28-day	18/47	ALC, CD3, CD4, CD8
Miao 2024	China	Retrospective; respiratory ICU; severe pneumonia with sepsis	Chinese emergency sepsis guideline (2018), consistent with Sepsis-3 framework	RICU days 1, 3, and 7; day 1 used	28-day	30/43	CD4
Dong 2025	China	Retrospective older-adult cohort; ICU	Sepsis-3	Worst value within first 24 h	30-day	140/345	ALC, CD3, CD4, CD8
Du 2025	China	Retrospective septic-shock cohort	Sepsis-3/septic-shock criteria	Admission day	28-day	40/61	CD4, CD8
Li (Front Immunol) 2025	China	Prospective multicenter older-adult ICU cohort	Sepsis-3	Within 24 h of ICU admission	28-day	158/1039	CD3*, CD4, CD8
Cheng 2025	China	Prospective single-center ICU cohort	Sepsis-3	First ICU day/within 24 h	In-hospital (**Table 3**; Methods states 28-day)	13/49	ALC, CD3, CD4

Non-survivor and survivor sample sizes are shown for the mortality endpoint used in each study. Sample sizes overlap across markers and should not be summed. *In Li et al. (Front Immunol) 2025, TLC denotes T-lymphocyte count and was assigned to the CD3 compartment. Detailed centers, recruitment periods, measurement methods, and adjusted analyses are provided in **Supplementary Tables 2**, **3**.

### 3.2 Primary analyses of CD4+ and CD8+ T-cell counts

The four primary analyses are summarized in **Table 2**. Thirteen studies reported absolute CD4+ T-cell counts, including 744 non-survivors and 2,595 survivors. CD4+ T-cell counts were lower in non-survivors (MD = -86.06 cells/µL, 95% CI -127.29 to -44.83, P < 0.001, I² = 77.0%, τ² = 3,259.22); the 95% prediction interval was -218.44 to 46.31 cells/µL, indicating that a future cohort could show a null association.

**Table 2:** Primary meta-analysis results for the four peripheral immune-cell markers.

Marker	k	MD (cells/µL)	95% CI	P value	I² (%)	τ²	95% prediction interval
CD4	13	-86.06	-127.29 to -44.83	<0.001	77.0	3,259.22	-218.44 to 46.31
CD8	11	-41.83	-68.37 to -15.29	0.0056	61.9	883.24	-114.26 to 30.60
CD3	10	-133.99	-220.41 to -47.56	0.0066	88.3	10,917.36	-390.53 to 122.56
ALC	10	-201.46	-323.04 to -79.88	0.0046	71.7	18,634.96	-539.77 to 136.85

MD was calculated as non-survivors minus survivors. Primary analyses used REML random-effects models with modified Hartung-Knapp 95% CIs. All 95% prediction intervals crossed zero. P values are two-sided.

**Figure 2:**
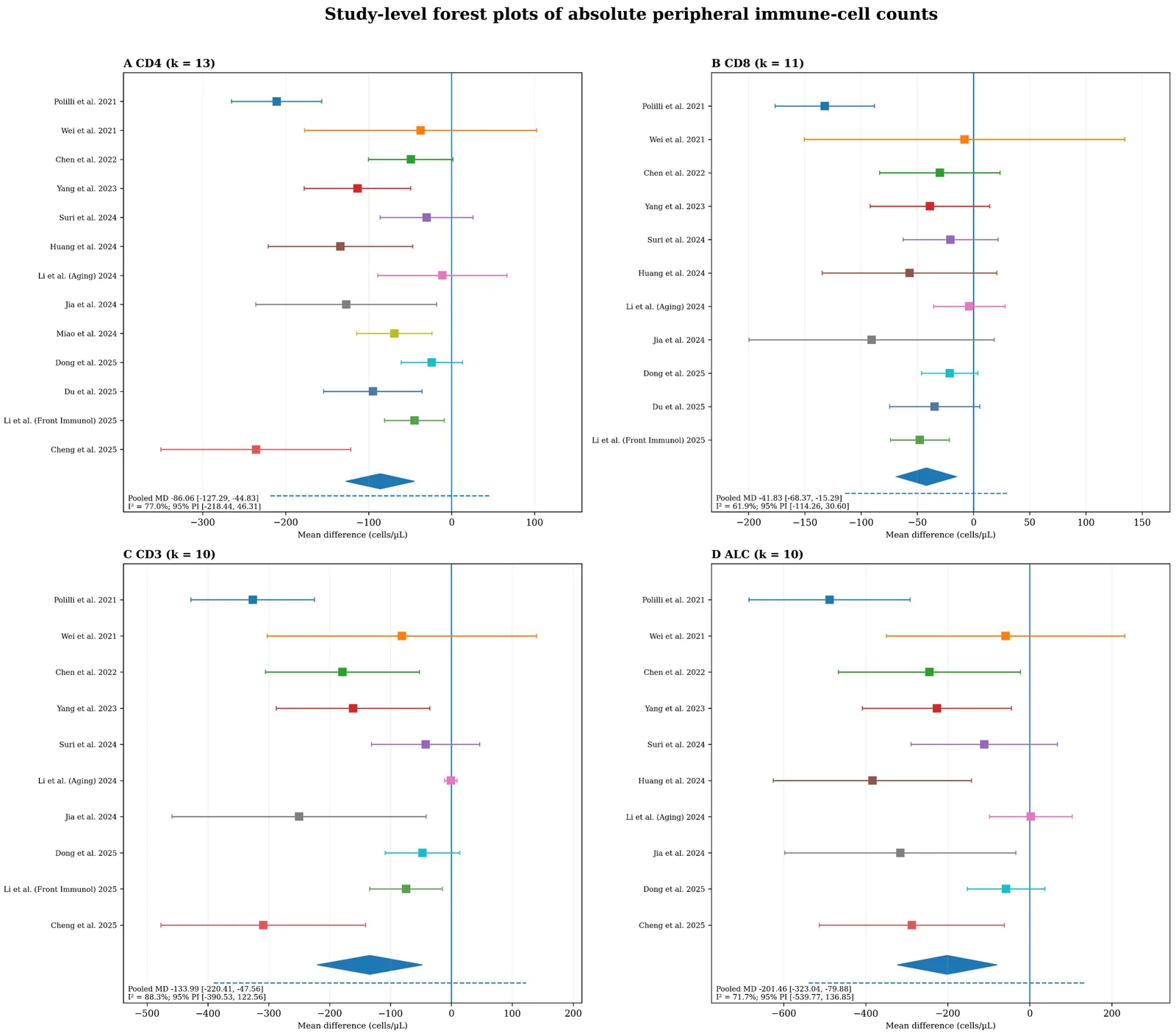
Study-level forest plots of absolute peripheral immune-cell counts. **(A)** CD4; **(B)** CD8; **(C)** CD3; **(D)** ALC. Individual squares and horizontal lines show study-specific mean differences and 95% CIs; diamonds show pooled REML estimates with modified Hartung-Knapp 95% CIs. Negative values indicate lower counts in non-survivors. Dashed horizontal segments below the pooled estimate show the 95% prediction interval; all four prediction intervals include zero.

Eleven studies reported absolute CD8+ T-cell counts, including 701 non-survivors and 2,503 survivors. CD8+ T-cell counts were lower in non-survivors (MD = -41.83 cells/µL, 95% CI -68.37 to -15.29, P = 0.0056, I² = 61.9%, τ² = 883.24); the 95% prediction interval was -114.26 to 30.60 cells/µL (**Figure 2**).

### 3.3 Supporting analyses of CD3+ T-cell counts and ALC

Ten studies reported absolute CD3+ T-cell counts, including 645 non-survivors and 2,420 survivors. CD3+ T-cell counts were lower in non-survivors (MD = -133.99 cells/µL, 95% CI -220.41 to -47.56, P = 0.0066, I² = 88.3%, τ² = 10,917.36); the 95% prediction interval was -390.53 to 122.56 cells/µL.

Ten studies reported ALC, including 516 non-survivors and 1,452 survivors. ALC was lower in non-survivors (MD = -201.46 cells/µL, 95% CI -323.04 to -79.88, P = 0.0046, I² = 71.7%, τ² = 18,634.96); the 95% prediction interval was -539.77 to 136.85 cells/µL. Thus, all four pooled averages were statistically different from zero, but every prediction interval included zero.

### 3.4 Sensitivity, relative-effect, and influence analyses

**Figure 3:**
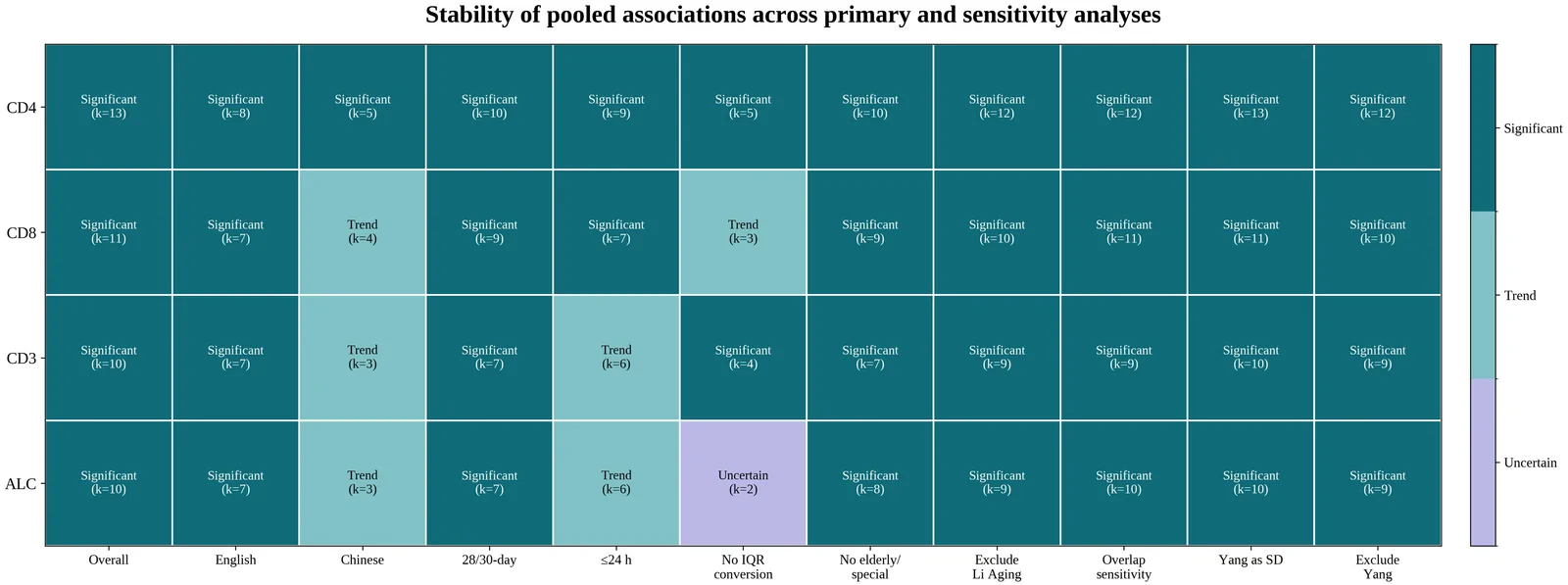
Stability of pooled associations across primary and sensitivity analyses. Each cell reports the qualitative result and number of contributing studies (k). “Significant” indicates MD < 0 with a 95% CI excluding zero; “Trend” indicates MD < 0 with a CI including zero; “Uncertain” indicates a sparse or non-informative estimate. Analyses with k ≤ 3 are interpreted cautiously.

CD4 remained significantly lower across English-language studies, Chinese-language studies, 28/30-day mortality, explicit ≤24-h sampling, exclusion of converted data, exclusion of older/special cohorts, exclusion of Li (Aging), alternative handling of Yang dispersion, and potential-overlap scenarios. For explicit ≤24-h sampling, the pooled MD was -65.86 cells/µL (95% CI -108.74 to -22.97; k = 9) (**Supplementary Figure 4**). In a *post hoc* risk-of-bias sensitivity analysis excluding the eight studies classified as high risk in the descriptive study-level QUIPS synthesis, CD4 remained significantly lower (k = 5; MD = -106.57, 95% CI -195.02 to -18.12). CD8 remained significant in most restrictions, including explicit ≤24-h sampling (MD = -28.45, 95% CI -48.74 to -8.15; k = 7), but exclusion of converted data left only three studies and produced a directional, non-significant estimate (MD = -44.96, 95% CI -95.88 to 5.97). After exclusion of descriptively high-risk studies, the CD8 estimate remained negative but imprecise (k = 4; MD = -60.97, 95% CI -145.90 to 23.95). CD3 and ALC remained negative after restriction to explicit ≤24-h sampling, but their CIs crossed zero; exclusion of converted data left four CD3 studies with a significant estimate and only two ALC studies with an uninformative interval. The lower-risk subset contained only two CD3 and three ALC studies, producing negative but highly imprecise estimates. Analyses with k ≤ 3 are treated as illustrative rather than confirmatory (**Table 3**, **Figure 3**, **Supplementary Table 4**, **Supplementary Figure 9**).

**Table 3:** Key restricted sensitivity analyses.

Marker	Analysis	k	MD	95% CI	P value	Interpretation
CD4	Overall	13	-86.06	-127.29 to -44.83	<0.001	Significantly lower
CD4	28/30-day mortality only	10	-62.84	-90.63 to -35.05	<0.001	Significantly lower
CD4	Explicit ≤24 h sampling only	9	-65.86	-108.74 to -22.97	0.0076	Significantly lower
CD4	Excluding converted median/IQR	5	-60.17	-92.34 to -28.01	0.0065	Significantly lower
CD4	Excluding elderly/special cohorts	10	-89.00	-134.88 to -43.11	0.0018	Significantly lower
CD4	Excluding descriptive overall high QUIPS risk	5	-106.57	-195.02 to -18.12	0.0287	Significantly lower
CD8	Overall	11	-41.83	-68.37 to -15.29	0.0056	Significantly lower
CD8	28/30-day mortality only	9	-30.35	-47.97 to -12.72	0.0041	Significantly lower
CD8	Explicit ≤24 h sampling only	7	-28.45	-48.74 to -8.15	0.014	Significantly lower
CD8	Excluding converted median/IQR	3	-44.96	-95.88 to 5.97	0.0628	Directional trend
CD8	Excluding elderly/special cohorts	9	-45.09	-81.06 to -9.13	0.0201	Significantly lower
CD8	Excluding descriptive overall high QUIPS risk	4	-60.97	-145.90 to 23.95	0.1065	Directional trend
CD3	Overall	10	-133.99	-220.41 to -47.56	0.0066	Significantly lower
CD3	28/30-day mortality only	7	-85.78	-162.19 to -9.37	0.0334	Significantly lower
CD3	Explicit ≤24 h sampling only	6	-95.03	-204.25 to 14.20	0.0756	Directional trend
CD3	Excluding converted median/IQR	4	-113.76	-217.70 to -9.83	0.04	Significantly lower
CD3	Excluding elderly/special cohorts	7	-140.93	-260.91 to -20.95	0.0283	Significantly lower
CD3	Excluding descriptive overall high QUIPS risk	2	-183.22	-1988.34 to 1621.90	0.4199	Uncertain
ALC	Overall	10	-201.46	-323.04 to -79.88	0.0046	Significantly lower
ALC	28/30-day mortality only	7	-157.03	-297.22 to -16.83	0.0337	Significantly lower
ALC	Explicit ≤24 h sampling only	6	-148.77	-313.59 to 16.04	0.068	Directional trend
ALC	Excluding converted median/IQR	2	-179.29	-1178.94 to 820.36	0.2632	Uncertain
ALC	Excluding elderly/special cohorts	8	-218.22	-369.33 to -67.10	0.0112	Significantly lower
ALC	Excluding descriptive overall high QUIPS risk	3	-322.14	-828.60 to 184.32	0.1116	Directional trend

k is displayed for every restriction. Analyses with k ≤ 3 are illustrative because τ² and small-sample uncertainty are difficult to estimate reliably. Full language, Yang-dispersion, cohort-overlap, and combined sensitivity analyses are reported in **Supplementary Table 4**.

Language-stratified estimates did not show opposite directions. CD4 was significant in both English- and Chinese-language subsets; Chinese-language estimates for CD8, CD3, and ALC were negative but imprecise (**Supplementary Table 4**, **Supplementary Figure 3**). Language meta-regression was not significant for CD4 (P = 0.491), CD8 (P = 0.414), CD3 (P = 0.524), or ALC (P = 0.277). Sampling-time meta-regression was also non-significant for all markers (P = 0.113-0.274), although these ecological analyses were underpowered (**Supplementary Table 7**). In 44 leave-one-out fits, every pooled MD remained negative with a 95% CI excluding zero (**Supplementary Table 11**, **Supplementary Figure 2**). Baujat diagnostics identified Polilli 2021 as the most consistent contributor to heterogeneity and influence across markers (**Supplementary Table 12**, **Supplementary Figure 7**).

**Figure 4:**
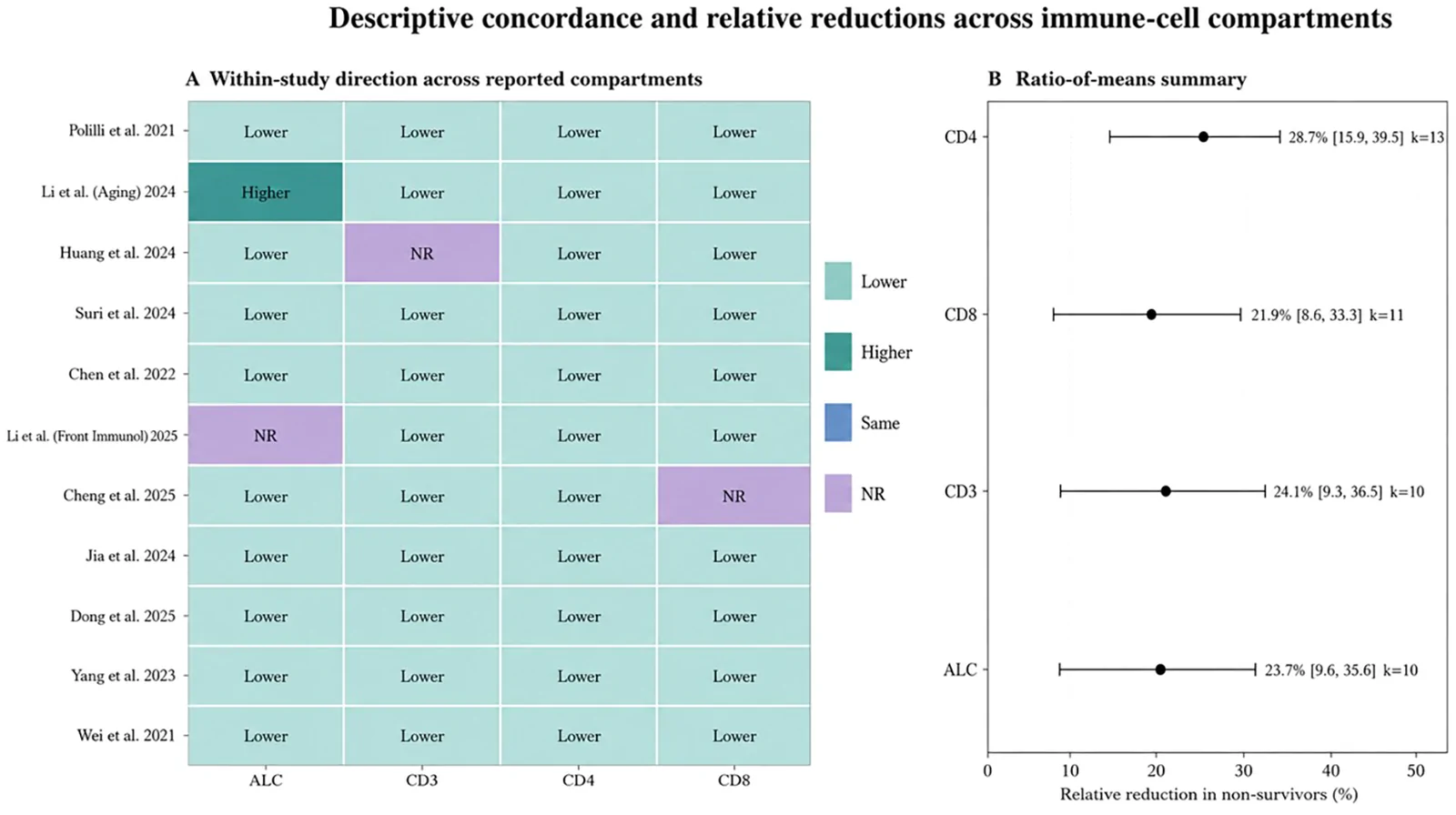
Descriptive concordance and relative-effect summaries. **(A)** Within-study direction across ALC, CD3, CD4, and CD8 for studies reporting at least three compartments. This *post hoc* display summarizes concordance among correlated measures. **(B)** Pooled relative reductions derived from ratio-of-means analyses. Confidence intervals overlap and no formal between-marker comparison was performed.

Among the 11 studies reporting at least three compartments, 10 showed lower counts in all reported compartments; Li et al. (Aging) showed lower CD3, CD4, and CD8 counts but a slightly higher ALC (**Figure 4A**, **Supplementary Table 8**). RoM analyses indicated mean ratios of 0.713 for CD4, 0.781 for CD8, 0.759 for CD3, and 0.763 for ALC, corresponding to relative reductions of 28.7%, 21.9%, 24.1%, and 23.7%, respectively. Confidence intervals overlapped, and no formal between-marker comparison was performed. In no-conversion RoM analyses, CD4 (RoM 0.802, 95% CI 0.688-0.935; k = 5) and CD3 (0.818, 0.687-0.975; k = 4) remained significant, whereas CD8 (0.787, 0.583-1.063; k = 3) and ALC (0.823, 0.185-3.652; k = 2) were imprecise (**Figure 4B**; **Supplementary Table 6**).

The Yang 2023 assumption did not change effect direction or statistical significance. Treating the printed dispersion directly as SD increased CD3 heterogeneity from I² = 88.3% to 97.8%; excluding Yang preserved the pooled direction. Potential overlap was not supported for Chen 2022 versus Li 2025 because recruitment periods did not overlap, and Huang 2024 was conducted at a different center. However, Li (Front Immunol) 2025 and Cheng 2025 shared Peking Union Medical College Hospital, ChiCTR2300074175, authors, and overlapping recruitment periods. Excluding the smaller Cheng cohort preserved CD4 (MD = -77.48, 95% CI -116.25 to -38.70) and CD3 (MD = -117.60, 95% CI -204.58 to -30.62); alternative exclusions yielded the same direction and significance. Actual patient duplication could not be established from published reports (**Supplementary Tables 9**, **10**, **Supplementary Figures 5**, **6**).

### 3.5 Small-study effects and exploratory meta-regression

Funnel plots and Egger tests did not indicate clear asymmetry for CD4 (P = 0.147) or CD8 (P = 0.473). Possible small-study effects were detected for CD3 (P = 0.0022) and ALC (P = 0.016), although the small number of studies and substantial heterogeneity limit interpretation. None of the seven study-level variables, including language and explicit ≤24-h sampling, was significant in univariable meta-regression. These analyses were exploratory and ecological (**Supplementary Table 7**, **Supplementary Figure 1**).

### 3.6 Risk of bias and evidence robustness

QUIPS assessment identified confounding and statistical analysis/reporting as the principal risks of bias. Seven of 13 studies were at high risk in the confounding domain, and four were at high risk in analysis/reporting. The descriptive overall synthesis classified eight studies as high risk and five as moderate risk; no study was globally low risk (**Supplementary Table 13**, **Supplementary Figure 8**). Eleven studies reported some multivariable analysis, but covariate sets and outcomes differed. Absolute CD4 remained associated in selected studies, whereas several studies—including the largest multicenter cohort—did not support an independent association for CD4 or total T-cell count after adjustment. Evidence for individual CD8, CD3, and ALC counts was similarly inconsistent (**Supplementary Table 3**). Across the robustness assessment, CD4 showed the most consistent group-level association: the pooled difference remained significant after exclusion of statistically converted studies and after exclusion of studies with a high descriptive overall QUIPS judgment. CD8 became imprecise after either of these restrictions; CD3 retained a significant no-conversion estimate but had high heterogeneity, unresolved Yang dispersion uncertainty, and only two lower-risk studies; and ALC became highly imprecise when converted or high-risk studies were excluded. All four 95% prediction intervals crossed zero, 11 of 13 studies were conducted in China, and possible small-study effects were detected for CD3 and ALC. These findings are summarized descriptively rather than as formal certainty grades (**Table 4**, **Supplementary Table 14**).

**Table 4:** Summary of evidence robustness and major limitations across peripheral immune-cell markers.

Marker	Main pooled association	95% prediction interval	Key robustness findings	Principal limitations	Descriptive interpretation
CD4	MD -86.06 cells/µL (95% CI -127.29 to -44.83)	-218.44 to 46.31	Significant after excluding converted data (k=5; MD -60.17) and after excluding high descriptive QUIPS-risk studies (k=5; MD -106.57)	8/13 contributing studies high descriptive QUIPS risk; 11/13 cohorts from China; adjusted evidence inconsistent	Most consistent group-level association; does not establish independent prognostic value
CD8	MD -41.83 cells/µL (95% CI -68.37 to -15.29)	-114.26 to 30.60	Negative in most restrictions	No-conversion analysis imprecise (k=3; 95% CI -95.88 to 5.97); lower-risk analysis imprecise (k=4; 95% CI -145.90 to 23.95); adjusted evidence inconsistent	Supportive group-level association but sensitive to data handling and risk-of-bias restriction
CD3	MD -133.99 cells/µL (95% CI -220.41 to -47.56)	-390.53 to 122.56	No-conversion analysis remained significant (k=4; MD -113.76)	High heterogeneity (I² 88.3%); Yang dispersion ambiguity; only two lower-risk studies; possible small-study effects	Association present, but heterogeneity and sparse lower-risk evidence limit robustness
ALC	MD -201.46 cells/µL (95% CI -323.04 to -79.88)	-539.77 to 136.85	Overall and leave-one-out estimates remained negative	No-conversion analysis based on two studies and uninformative; lower-risk analysis based on three studies and imprecise; possible small-study effects	Interpret cautiously; most sensitive to conversion and sparse lower-risk evidence

This table summarizes robustness of the pooled unadjusted survivor–non-survivor associations; it is not a formal certainty-of-evidence grade and does not establish independent prognostic performance. All 95% prediction intervals crossed zero. QUIPS, Quality In Prognosis Studies; MD, mean difference. Detailed marker-specific limitations are provided in **Supplementary Table 14**.

## 4 Discussion

### 4.1 Principal findings and biological interpretation of peripheral adaptive immune cell depletion

This review found lower mean peripheral CD4+, CD8+, and CD3+ T-cell counts and lower ALC among adults with sepsis who died than among survivors. The pooled direction was stable in leave-one-out analysis and many sensitivity analyses, with CD4 showing the greatest descriptive consistency. After exclusion of studies classified as high risk in the descriptive QUIPS synthesis, the CD4 difference remained statistically significant, whereas CD8, CD3, and ALC estimates remained negative but became imprecise as the evidence set contracted. All prediction intervals crossed zero, and adjusted analyses across individual studies were inconsistent. Taken together, the findings support a group-level association between lower circulating adaptive immune-cell counts and mortality, with the most consistent evidence for CD4 and greater uncertainty for CD8, CD3, and ALC because of data-conversion dependence, heterogeneity, or sparse lower-risk evidence.

The *post hoc* concordance display showed parallel within-study reductions across the related lymphocyte and T-cell compartments in most studies. Because these measurements are correlated, the display is best viewed as a compact summary of direction rather than as additional statistical evidence. Lower circulating counts may reflect apoptosis, redistribution, endothelial adhesion, tissue trafficking, reduced thymic output, inadequate homeostatic proliferation, or a combination of these processes. Pathological studies in fatal sepsis demonstrate adaptive immune-cell depletion together with impaired antigen presentation and inhibitory signaling (5, 6), but peripheral counts alone cannot identify the responsible mechanism.

Our analysis quantifies circulating compartment size rather than cellular competence. Absolute counts cannot measure antigen specificity, clonal diversity, proliferation, T-cell receptor signaling, cytokine production, cytotoxicity, or exhaustion. We therefore describe numerical lymphocyte/T-cell depletion and avoid inferring functional exhaustion or a reduced “immune reserve” as a directly measured construct.

### 4.2 CD4+ T-cell count as the most descriptively stable count-based marker

CD4+ T-cell counts were reported in all 13 studies and remained lower across the main and sensitivity analyses, including the *post hoc* exclusion of studies classified as high risk in the descriptive QUIPS synthesis. This consistency is descriptively notable and biologically plausible because CD4+ T cells coordinate antigen-driven responses, CD8+ expansion, B-cell help, and multiple helper and regulatory lineages. However, no formal head-to-head comparison among markers was performed, the RoM confidence intervals overlapped, and the lower-risk subset remained heterogeneous. Selected individual studies, including the largest multicenter cohort, also did not show independent CD4 effects after adjustment for clinical factors.

Recent single-cell multi-omic evidence illustrates this internal complexity. Ye et al. resolved adult CD4+ T cells into seven subsets and reported lower proportions of GZMK+ effector-memory, GNLY+ terminal effector-memory, and FOXP3+ regulatory subsets across several infection sources, whereas two central-memory CD4+ T-cell subsets displayed contrasting biological and prognostic associations (11). A GPR183+NR4A2+ central-memory subset was enriched in abdominal, pulmonary, and skin-source sepsis and exhibited reduced costimulatory molecules, increased inhibitory receptors, diminished effector-gene expression, enhanced stress and apoptotic programs, and oligoclonal expansion. CD4-specific manipulation of Nr4a2 also altered survival and bacterial clearance in murine sepsis models (11). These findings provide a mechanistic rationale for the coexistence of lower total CD4 counts and abnormal states within residual CD4 cells, while the causal animal data should not be directly extrapolated to adult clinical populations.

CD4 count may therefore serve as an accessible quantitative descriptor that can prompt further immune phenotyping. The current evidence does not yet establish a clinical cutoff, incremental predictive value beyond established severity scores, or a treatment-selection role.

### 4.3 Coexistence of numerical depletion and subset-specific remodeling within CD4+ and CD8+ T-cell compartments

Total-cell counts and subset composition operate at different levels of resolution and may move in apparently discordant directions. This is particularly relevant to regulatory T cells. Tregs may limit excessive inflammation early in sepsis, whereas sustained or excessive suppressive activity may impair effector responses and pathogen clearance (12). Importantly, an increased proportion of Tregs within the CD4 compartment does not necessarily indicate absolute Treg expansion. Previous studies have reported higher Treg proportions despite lower absolute Treg counts, with particularly low absolute counts in septic shock; in some cohorts, survivors had higher absolute Treg counts than non-survivors (38, 39). The lower total CD4 counts observed in our meta-analysis should therefore not be interpreted as evidence that Treg expansion is the cause of immunosuppression. Total CD4 counts, absolute Treg counts, relative Treg proportions, and suppressive function are distinct measurements that require separate interpretation.

A similar principle applies to CD8+ T cells. The overall CD8 MD was significant, but the result became non-significant when analyses were restricted to the three studies that did not require median/IQR conversion. This sensitivity reflects both limited information and the normality assumptions of the Luo/Wan transformations, which may be imperfect for right-skewed cell counts. Thus, the pooled CD8 association should be considered less secure than the CD4 association. At the biological level, contraction of the overall CD8 compartment can still coexist with selective expansion of inflammatory or antigen-driven clones (11).

These observations help reconcile the apparently opposing concepts of immune depletion and immune activation in sepsis. A shrinking total cellular pool may still contain subsets with intense inflammatory activity, inhibitory signaling, clonal expansion, or impaired effector function. The pooled absolute counts in the present study capture the size of the major circulating compartments, whereas single-cell and functional studies reveal how those compartments are internally remodeled. Both levels are necessary for a biologically coherent interpretation.

### 4.4 Biological and methodological sources of heterogeneity

Although all four pooled mean effects were statistically significant, heterogeneity was moderate to very high and every 95% prediction interval crossed zero. These intervals indicate that a future cohort may show a smaller, absent, or potentially reversed difference. Sensitivity analyses establish that the direction is not dependent on a single study, but they do not remove genuine variation in populations, measurements, or clinical contexts.

Neither language nor explicit ≤24-h sampling significantly modified the effect in exploratory meta-regression, but these null results have low power. Five of eight English-language articles were still Chinese cohorts, so language is not a proxy for geography. Eleven of 13 studies were conducted in China, and several were restricted to older adults, severe pneumonia, or septic shock. Regional treatment practices, case mix, corticosteroid or immunomodulator exposure, flow-cytometry platforms, counting methods, and laboratory reference ranges could systematically influence observed associations. Generalizability to non-Asian populations is therefore limited.

Biological heterogeneity is also expected in sepsis. Transcriptomic studies have identified host-response endotypes with differing adaptive immune activity and mortality risk (8, 9), while single-cell studies show that similar total cell counts may conceal distinct cellular compositions, clonotypes, and regulatory programs (10, 11). Infection source is another plausible determinant because abdominal, pulmonary, urinary, and other sources of sepsis are associated with different host responses and clinical trajectories (40, 41). Age and immunosenescence may further modify baseline lymphocyte reserve and recovery capacity. Ye et al. profiled peripheral blood from adults and children with different infection sources; the study did not directly compare blood with multiple tissues. Accordingly, the present findings support infection-source and age-related heterogeneity in circulating immune profiles, but they do not establish a direct correspondence between peripheral blood and tissue immunity.

### 4.5 Clinical implications: from accessible counts to stratified immune assessment

ALC and CD3+/CD4+/CD8+ counts are more accessible than transcriptomic or single-cell platforms, but the present meta-analysis demonstrates group-level differences rather than independent prognostic performance. Most pooled inputs were not adjusted for severity, and the largest study did not retain CD4 or T-cell count as independent predictors. Without standardized thresholds, serial measurements, individual-participant data, and incremental prediction analyses, no count-based cutoff can be recommended for clinical decision-making.

A more defensible translational pathway would use serial ALC and CD3+/CD4+/CD8+ counts as a first-layer screen for persistent or progressive quantitative immune-cell loss. Patients with sustained reductions could then undergo a second layer of mechanistic phenotyping, including absolute and relative Treg measures, monocytic HLA-DR, PD-1 and TIM-3 expression, Ki-67, ex vivo cytokine-stimulation assays, and T-cell receptor diversity (13, 14). Dynamic trajectories may be more informative than a single early measurement because they can distinguish transient stress-related lymphopenia from persistent depletion or failure of immune recovery. Elevated PD-1 expression has been associated with mortality, nosocomial infection, and immune dysfunction in septic shock, further illustrating the potential value of combining quantitative and functional markers (42).

This tiered framework is also relevant to immunotherapy. Interleukin-7 can increase circulating lymphocyte counts in patients with septic shock and lymphopenia, but available trials do not establish a reduction in mortality or secondary infection (15). Early checkpoint-inhibitor studies have primarily demonstrated safety and pharmacodynamic feasibility rather than definitive clinical benefit (43). Immune-cell counts may therefore contribute to patient enrichment, but treatment decisions will require evidence that identifies the appropriate immune phenotype, therapeutic window, and expected mechanism of benefit.

### 4.6 Limitations and future directions

Several limitations are central to interpretation. First, the pooled MDs are unadjusted observational contrasts and cannot distinguish an independent prognostic factor from a downstream marker of disease severity or reverse causation; adjusted results were inconsistent. Second, 11 of 13 cohorts were Chinese, limiting extrapolation to other health systems and populations. Third, sepsis definitions, infection sources, mortality windows, and sampling anchors varied, and most studies relied on a single early measurement of a dynamic marker. Restriction to explicit ≤24-h sampling reduced precision for CD3 and ALC. Fourth, 55%-80% of contributing studies required median/IQR conversion, and no-conversion analyses left only three CD8 studies and two ALC studies. Fifth, flow-cytometry platforms, antibody panels, gating, and absolute-count methods were incompletely standardized. Sixth, Yang 2023 contained unresolved SD-versus-SE ambiguity that materially affected CD3 heterogeneity; author clarification was unavailable at the time of revision. Seventh, patient overlap between Li (Front Immunol) and Cheng could not be excluded, although mutually exclusive removal analyses were stable. Eighth, the *post hoc* exclusion of studies classified as high risk in the descriptive QUIPS synthesis left only five CD4, four CD8, two CD3, and three ALC studies; the analysis supported the direction of the findings but could not remove residual bias or provide precise estimates for most markers. Ninth, CD3 and ALC may be affected by small-study effects. Finally, the hierarchical analysis was *post hoc*, mathematically non-independent, and purely descriptive.

Future studies should standardize flow-cytometric acquisition, gating, units, and reporting and should obtain repeated measurements at admission, 24 h, day 3, and day 7. Such designs could distinguish transient lymphopenia, persistent low counts, and failed immune recovery. Individual-participant-data meta-analysis is needed to examine modification by infection source, pathogen, age, shock status, comorbidity, and treatment exposure and to determine whether immune-cell trajectories add predictive value beyond clinical severity scores. Higher-resolution prospective studies should integrate absolute counts with CITE-seq, single-cell ATAC sequencing, T-cell receptor sequencing, and functional stimulation assays. This combined approach may clarify when numerical deficiency is clinically meaningful, when it coexists with subset-specific activation or dysfunction, and which patients might benefit from targeted immune-restorative strategies.

## 5 Conclusion

Among adults with sepsis, non-survivors had lower mean ALC and CD3+, CD4+, and CD8+ T-cell counts than survivors. Evidence was most consistent for CD4, while CD8 and ALC estimates were more dependent on statistically converted data. The association was most consistent for CD4, whereas CD8, CD3, and ALC were more sensitive to data conversion, heterogeneity, or sparse lower-risk evidence. Substantial heterogeneity, geographic concentration, and prediction intervals crossing zero mean that these counts should not yet be used as standalone prognostic biomarkers. Standardized longitudinal studies with severity-adjusted analyses and functional validation are needed.

## Data Availability

The original contributions presented in the study are included in the article/**Supplementary Material**. Further inquiries can be directed to the corresponding author.
