# Supplementary material for: Peripheral adaptive immune cell counts and mortality in adult sepsis: a systematic review and meta-analysis
Source: Front Immunol. 2026 Sep 1;17:1937759. doi: 10.3389/fimmu.2026.1937759 (PMC13574945; doi:10.3389/fimmu.2026.1937759)
Supplement: Supplementary file 1 [file Table1.docx]

Supplementary Material

**Peripheral adaptive immune cell counts and mortality in adult sepsis: a systematic review and meta-analysis**

PROSPERO: CRD420261446443

## **Supplementary Table S1. Search strategies and records retrieved in the updated search through 7 August 2026**

| **Database** | **Search strategy** | **Records retrieved** |
| --- | --- | --- |
| PubMed | ("Sepsis"[Mesh] OR sepsis[tiab] OR "septic shock"[tiab] OR septicemia[tiab] OR septicaemia[tiab]) AND ("T-Lymphocyte Subsets"[Mesh] OR "CD4-Positive T-Lymphocytes"[Mesh] OR "CD8-Positive T-Lymphocytes"[Mesh] OR "Lymphocyte Count"[Mesh] OR "CD4 Lymphocyte Count"[Mesh] OR "Lymphopenia"[Mesh] OR CD3[tiab] OR CD4[tiab] OR CD8[tiab] OR "T lymphocyte"[tiab] OR "T lymphocytes"[tiab] OR T-lymphocyte*[tiab] OR "T cell"[tiab] OR "T cells"[tiab] OR "lymphocyte subset"[tiab] OR "lymphocyte subsets"[tiab] OR "T lymphocyte subset"[tiab] OR "T lymphocyte subsets"[tiab] OR "T-cell subset"[tiab] OR "T-cell subsets"[tiab] OR "lymphocyte count"[tiab] OR "lymphocyte counts"[tiab] OR "absolute lymphocyte count"[tiab] OR "absolute lymphocyte counts"[tiab] OR "absolute cell count"[tiab] OR "absolute cell counts"[tiab] OR lymphopenia[tiab] OR lymphocytopenia[tiab]) AND (mortality[tiab] OR death[tiab] OR fatalit*[tiab] OR survival[tiab] OR prognos*[tiab] OR outcome*[tiab] OR predict*[tiab]) | 163 |
| EMBASE | ('sepsis'/exp OR 'septic shock'/exp OR sepsis:ti,ab OR 'septic shock':ti,ab OR septicemia:ti,ab OR septicaemia:ti,ab) AND ('t lymphocyte subset'/exp OR 'cd4 positive t lymphocyte'/exp OR 'cd8 positive t lymphocyte'/exp OR 'lymphocyte count'/exp OR 'lymphopenia'/exp OR CD3:ti,ab OR CD4:ti,ab OR CD8:ti,ab OR 't lymphocyte*':ti,ab OR 't cell*':ti,ab OR 'lymphocyte subset*':ti,ab OR 't lymphocyte subset*':ti,ab OR 't cell subset*':ti,ab OR 'lymphocyte count*':ti,ab OR 'absolute lymphocyte count*':ti,ab OR 'absolute cell count*':ti,ab OR lymphopenia:ti,ab OR lymphocytopenia:ti,ab) AND (mortality:ti,ab OR death:ti,ab OR fatalit*:ti,ab OR survival:ti,ab OR prognos*:ti,ab OR outcome*:ti,ab OR predict*:ti,ab) | 462 |
| Web of Science Core Collection | TS=(sepsis OR "septic shock" OR septicemia OR septicaemia) AND TS=(CD3 OR CD4 OR CD8 OR "T lymphocyte*" OR "T-lymphocyte*" OR "T cell*" OR "lymphocyte subset*" OR "T lymphocyte subset*" OR "T-cell subset*" OR "lymphocyte count*" OR "absolute lymphocyte count*" OR "absolute cell count*" OR lymphopenia OR lymphocytopenia) AND TS=(mortality OR death OR fatalit* OR survival OR prognos* OR outcome* OR predict*) | 741 |
| Cochrane Library | (sepsis OR "septic shock" OR septicemia OR septicaemia):ti,ab,kw AND (CD3 OR CD4 OR CD8 OR "T lymphocyte" OR "T lymphocytes" OR "T cell" OR "T cells" OR "lymphocyte subset" OR "lymphocyte subsets" OR "T lymphocyte subset" OR "T cell subset" OR "lymphocyte count" OR "absolute lymphocyte count" OR "absolute cell count" OR lymphopenia OR lymphocytopenia):ti,ab,kw AND (mortality OR death OR fatality OR survival OR prognosis OR outcome OR prediction OR predict):ti,ab,kw | 287 |
| CNKI | 主题=(脓毒症 OR 脓毒性休克 OR 败血症) AND 主题=(CD3 OR CD4 OR CD8 OR T淋巴细胞 OR T细胞 OR 淋巴细胞亚群 OR T淋巴细胞亚群 OR T细胞亚群 OR 淋巴细胞计数 OR 绝对淋巴细胞计数 OR 绝对计数 OR 淋巴细胞减少 OR 淋巴细胞缺乏) AND 主题=(死亡 OR 病死率 OR 生存 OR 预后 OR 结局 OR 预测) | 937 |
| Wanfang Data | 主题=(脓毒症 OR 脓毒性休克 OR 败血症) AND 主题=(CD3 OR CD4 OR CD8 OR T淋巴细胞 OR T细胞 OR 淋巴细胞亚群 OR T淋巴细胞亚群 OR T细胞亚群 OR 淋巴细胞计数 OR 绝对淋巴细胞计数 OR 绝对计数 OR 淋巴细胞减少 OR 淋巴细胞缺乏) AND 主题=(死亡 OR 病死率 OR 生存 OR 预后 OR 结局 OR 预测) | 1,034 |
| VIP | 主题=(脓毒症 OR 脓毒性休克 OR 败血症) AND 主题=(CD3 OR CD4 OR CD8 OR T淋巴细胞 OR T细胞 OR 淋巴细胞亚群 OR T淋巴细胞亚群 OR T细胞亚群 OR 淋巴细胞计数 OR 绝对淋巴细胞计数 OR 绝对计数 OR 淋巴细胞减少 OR 淋巴细胞缺乏) AND 主题=(死亡 OR 病死率 OR 生存 OR 预后 OR 结局 OR 预测) | 844 |
| SinoMed | ("脓毒症"[主题] OR "脓毒性休克"[主题] OR "败血症"[主题]) AND ("CD3"[常用字段] OR "CD4"[常用字段] OR "CD8"[常用字段] OR "T淋巴细胞"[常用字段] OR "T细胞"[常用字段] OR "淋巴细胞亚群"[常用字段] OR "T淋巴细胞亚群"[常用字段] OR "T细胞亚群"[常用字段] OR "淋巴细胞计数"[常用字段] OR "绝对淋巴细胞计数"[常用字段] OR "绝对计数"[常用字段] OR "淋巴细胞减少"[常用字段] OR "淋巴细胞缺乏"[常用字段]) AND ("死亡"[常用字段] OR "病死率"[常用字段] OR "生存"[常用字段] OR "预后"[常用字段] OR "结局"[常用字段] OR "预测"[常用字段]) | 782 |
| Chinese Medical Journal Full-text Database | 主题=(脓毒症 OR 脓毒性休克 OR 败血症) AND 主题=(CD3 OR CD4 OR CD8 OR T淋巴细胞 OR T细胞 OR 淋巴细胞亚群 OR T淋巴细胞亚群 OR T细胞亚群 OR 淋巴细胞计数 OR 绝对淋巴细胞计数 OR 绝对计数 OR 淋巴细胞减少 OR 淋巴细胞缺乏) AND 主题=(死亡 OR 病死率 OR 生存 OR 预后 OR 结局 OR 预测) | 396 |

## **Supplementary Table S2. Detailed characteristics, centers, recruitment periods, definitions, and sampling anchors**

| **Study** | **Country** | **Design/setting** | **Center and recruitment period** | **Sepsis definition** | **Sampling anchor** | **Mortality endpoint** | **Non-survivor/survivor n** | **Markers** |
| --- | --- | --- | --- | --- | --- | --- | --- | --- |
| Polilli 2021 | Italy | Retrospective cohort; infectious-disease ward | Pescara General Hospital; Jan 2015-Aug 2018 | Sepsis-2/ICD-9-CM-based ascertainment | Hospital entry; exact hours not reported | In-hospital | 49/229 | ALC, CD3, CD4, CD8 |
| Wei 2021 | China | Prospective observational; ICU | First Hospital of Shanxi Medical University; Dec 2019-Dec 2020 | Sepsis-3 | Day 1 (primary) and day 3 | 28-day | 18/44 | ALC, CD3, CD4, CD8 |
| Chen 2022 | China | Prospective observational; ICU | Peking Union Medical College Hospital; Apr 2020-Dec 2021 | Sepsis-3-based ICU cohort | ICU day 1 | 28-day | 13/68 | ALC, CD3, CD4, CD8 |
| Yang 2023 | China | Retrospective; surgical ICU | First Affiliated Hospital of Zhengzhou University; Jan 2020-Dec 2021 | Sepsis-3 | Morning after SICU admission | 28-day | 81/198 | ALC, CD3, CD4, CD8 |
| Suri 2024 | India | Prospective observational; respiratory ICU | AIIMS New Delhi; Mar 2018-Sep 2019 | Sepsis-3 | Enrollment within 48 h of arrival; baseline used | In-hospital | 41/59 | ALC, CD3, CD4, CD8 |
| Huang 2024 | China | Prospective observational; ICU/emergency ICU | Guangdong Provincial People's Hospital; Jun 2016-Sep 2022 | Sepsis-3 | Within 24 h of enrollment | 28-day | 29/71 | ALC, CD4, CD8 |
| Li (Aging) 2024 | China | Retrospective; ICU | First Affiliated Hospital, Hengyang Medical School, University of South China; Jun 2021-Jul 2023 | Sepsis-3 | Admission day 1 (primary) and day 3 | 28-day | 114/342 | ALC, CD3, CD4, CD8 |
| Jia 2024 | China | Retrospective; emergency department | Peking University First Hospital; Aug 2021-Dec 2022 | Sepsis-3 | Day 1 or 2 after recognition | 28-day | 18/47 | ALC, CD3, CD4, CD8 |
| Miao 2024 | China | Retrospective; respiratory ICU; severe pneumonia with sepsis | Zhengzhou Central Hospital; Jan 2022-Jun 2023 | Chinese emergency sepsis guideline (2018), consistent with Sepsis-3 framework | RICU days 1, 3, and 7; day 1 used | 28-day | 30/43 | CD4 |
| Dong 2025 | China | Retrospective older-adult cohort; ICU | First Affiliated Hospital of Wenzhou Medical University; Sep 2021-Sep 2023 | Sepsis-3 | Worst value within first 24 h | 30-day | 140/345 | ALC, CD3, CD4, CD8 |
| Du 2025 | China | Retrospective septic-shock cohort | Jinhua People's Hospital; Mar 2021-Apr 2024 | Sepsis-3/septic-shock criteria | Admission day | 28-day | 40/61 | CD4, CD8 |
| Li (Front Immunol) 2025 | China | Prospective multicenter older-adult ICU cohort | PUMCH, Beijing Shijitan, Beijing Jishuitan, and Beijing Hospital; Methods: Jun 2023-Dec 2024 | Sepsis-3 | Within 24 h of ICU admission | 28-day | 158/1039 | CD3*, CD4, CD8 |
| Cheng 2025 | China | Prospective single-center ICU cohort | Peking Union Medical College Hospital; Jan-Jul 2024 | Sepsis-3 | First ICU day/within 24 h | In-hospital (Table 3; Methods states 28-day) | 13/49 | ALC, CD3, CD4 |

## **Supplementary Table S3. Study-level adjusted evidence**

| **Study** | **Adjusted analysis** | **Covariates/strategy** | **Adjusted finding for absolute counts** |
| --- | --- | --- | --- |
| Polilli 2021 | Yes | Age, sex, CCI, qSOFA, PCT, platelets and other candidates | Continuous CD4 and CD4 <400 cells/µL remained associated with in-hospital mortality. |
| Wei 2021 | No | - | Only group comparisons, correlations, and ROC analyses. |
| Chen 2022 | Yes | Immune variables; clinical severity adjustment incompletely reported | Absolute T-cell, CD4, and CD8 counts did not remain independently associated; functional CD4 phenotypes did. |
| Yang 2023 | Yes | Lymphocyte-subset variables; no SOFA/APACHE II adjustment | ALC, CD3, and CD4 were not independently associated. |
| Suri 2024 | Yes | Variables with univariable P<0.10 | Baseline absolute ALC/CD3/CD4/CD8 counts were not independently associated; selected activation percentages and day-5 lymphocyte percentage were. |
| Huang 2024 | Yes | Shock, heart rate, lactate, CD4, CD8 and other candidates | CD4 remained associated; CD8 did not. |
| Li (Aging) 2024 | Yes | Clinical variables and a composite peripheral lymphocyte-subset score | The composite score, not an individual count, was independently associated. |
| Jia 2024 | Yes | ALC, CD3, CD4, CD8 and immune-subset variables | ALC and naive CD4 percentage remained associated; CD3/CD4/CD8 did not. |
| Miao 2024 | Yes | CURB-65, APACHE II, LAR, IL-6, CD4 | Day-1 CD4 (used in this meta-analysis) was not independently associated; later measurements were. |
| Dong 2025 | Yes | BMI, IL-6, bilirubin, lactate, invasive ventilation and others | Lymphocyte/T-cell counts were not retained in the final multivariable model. |
| Du 2025 | Yes | CRP, CD4, SOFA, acute kidney injury | CD4 remained associated with 28-day mortality. |
| Li (Front Immunol) 2025 | Yes | FI-lab, APACHE II, heart rate, pulmonary infection and lymphocyte subsets | T-cell count and CD4 were not independently associated; NK-cell count was retained. |
| Cheng 2025 | No | - | No multivariable mortality model. |

## **Supplementary Table S4. Complete sensitivity-analysis results**

| **Marker** | **Analysis** | **k** | **MD** | **95% CI** | **P** | **I² (%)** | **95% PI** | **Classification** |
| --- | --- | --- | --- | --- | --- | --- | --- | --- |
| CD4 | Overall | 13 | -86.06 | -127.29 to -44.83 | <0.001 | 77.0 | -218.44 to 46.31 | Significant |
| CD4 | English-language studies | 8 | -99.47 | -169.52 to -29.41 | 0.0121 | 83.6 | -295.25 to 96.32 | Significant |
| CD4 | Chinese-language studies | 5 | -67.26 | -119.60 to -14.93 | 0.0234 | 49.6 | -177.57 to 43.04 | Significant |
| CD4 | 28/30-day mortality only | 10 | -62.84 | -90.63 to -35.05 | <0.001 | 37.7 | -122.02 to -3.66 | Significant |
| CD4 | Explicit ≤24 h sampling only | 9 | -65.86 | -108.74 to -22.97 | 0.0076 | 58.4 | -158.41 to 26.70 | Significant |
| CD4 | Excluding converted median/IQR | 5 | -60.17 | -92.34 to -28.01 | 0.0065 | 0.0 | -97.05 to -23.30 | Significant |
| CD4 | Excluding elderly/special cohorts | 10 | -89.00 | -134.88 to -43.11 | 0.0018 | 72.8 | -218.88 to 40.88 | Significant |
| CD4 | Excluding Li Aging 2024 | 12 | -91.86 | -134.66 to -49.05 | <0.001 | 77.9 | -225.83 to 42.12 | Significant |
| CD8 | Overall | 11 | -41.83 | -68.37 to -15.29 | 0.0056 | 61.9 | -114.26 to 30.60 | Significant |
| CD8 | English-language studies | 7 | -50.26 | -94.44 to -6.08 | 0.0318 | 74.8 | -161.92 to 61.39 | Significant |
| CD8 | Chinese-language studies | 4 | -26.48 | -58.35 to 5.38 | 0.0773 | 0.0 | -69.56 to 16.60 | Directional trend |
| CD8 | 28/30-day mortality only | 9 | -30.35 | -47.97 to -12.72 | 0.0041 | 0.0 | -56.65 to -4.04 | Significant |
| CD8 | Explicit ≤24 h sampling only | 7 | -28.45 | -48.74 to -8.15 | 0.014 | 0.0 | -60.53 to 3.64 | Significant |
| CD8 | Excluding converted median/IQR | 3 | -44.96 | -95.88 to 5.97 | 0.0628 | 0.0 | -195.33 to 105.42 | Directional trend |
| CD8 | Excluding elderly/special cohorts | 9 | -45.09 | -81.06 to -9.13 | 0.0201 | 66.6 | -137.07 to 46.88 | Significant |
| CD8 | Excluding Li Aging 2024 | 10 | -47.11 | -75.13 to -19.09 | 0.0042 | 57.7 | -118.72 to 24.49 | Significant |
| CD3 | Overall | 10 | -133.99 | -220.41 to -47.56 | 0.0066 | 88.3 | -390.53 to 122.56 | Significant |
| CD3 | English-language studies | 7 | -154.02 | -280.78 to -27.26 | 0.0249 | 91.4 | -498.98 to 190.94 | Significant |
| CD3 | Chinese-language studies | 3 | -84.20 | -264.83 to 96.44 | 0.1828 | 21.8 | -862.94 to 694.54 | Directional trend |
| CD3 | 28/30-day mortality only | 7 | -85.78 | -162.19 to -9.37 | 0.0334 | 77.4 | -266.75 to 95.19 | Significant |
| CD3 | Explicit ≤24 h sampling only | 6 | -95.03 | -204.25 to 14.20 | 0.0756 | 82.1 | -358.35 to 168.30 | Directional trend |
| CD3 | Excluding converted median/IQR | 4 | -113.76 | -217.70 to -9.83 | 0.04 | 3.7 | -315.14 to 87.62 | Significant |
| CD3 | Excluding elderly/special cohorts | 7 | -140.93 | -260.91 to -20.95 | 0.0283 | 89.8 | -454.83 to 172.96 | Significant |
| CD3 | Excluding Li Aging 2024 | 9 | -153.31 | -242.74 to -63.89 | 0.0042 | 76.5 | -399.99 to 93.36 | Significant |
| ALC | Overall | 10 | -201.46 | -323.04 to -79.88 | 0.0046 | 71.7 | -539.77 to 136.85 | Significant |
| ALC | English-language studies | 7 | -244.90 | -418.18 to -71.63 | 0.0135 | 78.0 | -683.24 to 193.43 | Significant |
| ALC | Chinese-language studies | 3 | -106.02 | -361.14 to 149.11 | 0.2157 | 24.4 | -1188.95 to 976.92 | Directional trend |
| ALC | 28/30-day mortality only | 7 | -157.03 | -297.22 to -16.83 | 0.0337 | 62.1 | -487.26 to 173.20 | Significant |
| ALC | Explicit ≤24 h sampling only | 6 | -148.77 | -313.59 to 16.04 | 0.068 | 64.9 | -535.05 to 237.51 | Directional trend |
| ALC | Excluding converted median/IQR | 2 | -179.29 | -1178.94 to 820.36 | 0.2632 | 0.0 | Not estimated | Uncertain |
| ALC | Excluding elderly/special cohorts | 8 | -218.22 | -369.33 to -67.10 | 0.0112 | 74.1 | -607.72 to 171.29 | Significant |
| ALC | Excluding Li Aging 2024 | 9 | -231.11 | -353.47 to -108.76 | 0.0024 | 63.9 | -538.72 to 76.50 | Significant |
| CD4 | Preferred overlap sensitivity | 12 | -77.48 | -116.25 to -38.70 | 0.0011 | 75.1 | -197.48 to 42.53 | Significant |
| CD8 | Preferred overlap sensitivity | 11 | -41.83 | -68.37 to -15.29 | 0.0056 | 61.9 | -114.26 to 30.60 | Significant |
| CD3 | Preferred overlap sensitivity | 9 | -117.60 | -204.58 to -30.62 | 0.0143 | 87.7 | -365.03 to 129.83 | Significant |
| ALC | Preferred overlap sensitivity | 10 | -201.46 | -323.04 to -79.88 | 0.0046 | 71.7 | -539.77 to 136.85 | Significant |
| CD4 | Yang printed values used as SD | 13 | -86.72 | -127.48 to -45.95 | <0.001 | 84.2 | -217.88 to 44.45 | Significant |
| CD8 | Yang printed values used as SD | 11 | -41.44 | -66.08 to -16.80 | 0.0038 | 62.0 | -109.75 to 26.87 | Significant |
| CD3 | Yang printed values used as SD | 10 | -134.62 | -218.31 to -50.92 | 0.0054 | 97.8 | -386.30 to 117.06 | Significant |
| ALC | Yang printed values used as SD | 10 | -201.98 | -318.42 to -85.53 | 0.0035 | 78.8 | -530.48 to 126.52 | Significant |
| CD4 | Yang 2023 excluded | 12 | -84.01 | -129.16 to -38.86 | 0.0018 | 78.2 | -225.20 to 57.19 | Significant |
| CD8 | Yang 2023 excluded | 10 | -42.39 | -72.14 to -12.65 | 0.0104 | 65.7 | -122.46 to 37.68 | Significant |
| CD3 | Yang 2023 excluded | 9 | -132.42 | -229.90 to -34.95 | 0.014 | 88.8 | -413.80 to 148.95 | Significant |
| ALC | Yang 2023 excluded | 9 | -200.63 | -338.73 to -62.54 | 0.0101 | 74.0 | -576.57 to 175.30 | Significant |
| CD4 | Combined: Yang used as SD + exclude Cheng | 12 | -78.68 | -116.77 to -40.59 | <0.001 | 84.6 | -198.36 to 41.00 | Significant |
| CD8 | Combined: Yang used as SD + exclude Cheng | 11 | -41.44 | -66.08 to -16.80 | 0.0038 | 62.0 | -109.75 to 26.87 | Significant |
| CD3 | Combined: Yang used as SD + exclude Cheng | 9 | -119.28 | -203.41 to -35.14 | 0.0114 | 98.0 | -362.70 to 124.15 | Significant |
| ALC | Combined: Yang used as SD + exclude Cheng | 10 | -201.98 | -318.42 to -85.53 | 0.0035 | 78.8 | -530.48 to 126.52 | Significant |
| CD4 | Combined: exclude Yang + exclude Cheng | 11 | -74.25 | -116.42 to -32.08 | 0.0028 | 76.3 | -201.15 to 52.65 | Significant |
| CD8 | Combined: exclude Yang + exclude Cheng | 10 | -42.39 | -72.14 to -12.65 | 0.0104 | 65.7 | -122.46 to 37.68 | Significant |
| CD3 | Combined: exclude Yang + exclude Cheng | 8 | -113.60 | -212.43 to -14.77 | 0.0299 | 88.2 | -386.82 to 159.61 | Significant |
| ALC | Combined: exclude Yang + exclude Cheng | 9 | -200.63 | -338.73 to -62.54 | 0.0101 | 74.0 | -576.57 to 175.30 | Significant |
| CD4 | Excluding studies with a high overall descriptive QUIPS judgment | 5 | -106.57 | -195.02 to -18.12 | 0.0287 | 83.6 | -334.55 to 121.40 | Significantly lower |
| CD8 | Excluding studies with a high overall descriptive QUIPS judgment | 4 | -60.97 | -145.90 to 23.95 | 0.1065 | 80.2 | -292.09 to 170.15 | Directional trend |
| CD3 | Excluding studies with a high overall descriptive QUIPS judgment | 2 | -183.22 | -1988.34 to 1621.90 | 0.4199 | 94.1 | NA | Uncertain |
| ALC | Excluding studies with a high overall descriptive QUIPS judgment | 3 | -322.14 | -828.60 to 184.32 | 0.1116 | 75.9 | -3001.02 to 2356.74 | Directional trend |

## **Supplementary Table S5. Full-text exclusion categories**

| **Reason** | **Number** |
| --- | --- |
| Continuous grouped absolute-count data unavailable or ineligible | 35 |
| Outcome/grouping ineligible | 11 |
| Design, population, or topic ineligible | 6 |
| Duplicate record or publication | 6 |
| Total | 58 |

## **Supplementary Table S6. Ratio-of-means analyses and no-conversion sensitivity**

| **Marker** | **Analysis** | **k** | **RoM** | **95% CI** | **P** | **Relative reduction** |
| --- | --- | --- | --- | --- | --- | --- |
| CD4 | Overall | 13 | 0.713 | 0.605 to 0.841 | <0.001 | 28.7% |
| CD4 | Excluding converted median/IQR | 5 | 0.802 | 0.688 to 0.935 | 0.0161 | 19.8% |
| CD8 | Overall | 11 | 0.781 | 0.667 to 0.914 | 0.0056 | 21.9% |
| CD8 | Excluding converted median/IQR | 3 | 0.787 | 0.583 to 1.063 | 0.0756 | 21.3% |
| CD3 | Overall | 10 | 0.759 | 0.635 to 0.907 | 0.0067 | 24.1% |
| CD3 | Excluding converted median/IQR | 4 | 0.818 | 0.687 to 0.975 | 0.0358 | 18.2% |
| ALC | Overall | 10 | 0.763 | 0.644 to 0.904 | 0.0057 | 23.7% |
| ALC | Excluding converted median/IQR | 2 | 0.823 | 0.185 to 3.652 | 0.3451 | 17.7% |

Log ratios of means and delta-method variances were pooled using REML with the same modified Hartung-Knapp adjustment as the mean-difference analyses. Prediction intervals were not calculated for this secondary analysis.

## **Supplementary Table S7. Egger tests and exploratory univariable meta-regression**

| **Marker** | **Analysis/moderator** | **k** | **Coefficient** | **SE** | **P** |
| --- | --- | --- | --- | --- | --- |
| CD4 | Egger intercept | 13 | -2.371 | 1.521 | 0.1473 |
| CD8 | Egger intercept | 11 | -0.898 | 1.199 | 0.4731 |
| CD3 | Egger intercept | 10 | -2.824 | 0.638 | 0.0022 |
| ALC | Egger intercept | 10 | -3.362 | 1.105 | 0.016 |
| CD4 | language_english | 13 | -28.269 | 39.642 | 0.4906 |
| CD4 | mortality_28_30 | 13 | 78.615 | 40.709 | 0.0796 |
| CD4 | converted_iqr | 13 | -36.374 | 38.894 | 0.3698 |
| CD4 | total_n_z | 13 | 18.920 | 18.217 | 0.3213 |
| CD4 | adverse_rate_z | 13 | 13.660 | 18.931 | 0.4856 |
| CD4 | elderly_cohort | 13 | 10.257 | 45.999 | 0.8276 |
| CD4 | explicit_within_24h | 13 | 49.896 | 39.929 | 0.2374 |
| CD8 | language_english | 11 | -20.476 | 23.898 | 0.4138 |
| CD8 | mortality_28_30 | 11 | 42.864 | 25.460 | 0.1266 |
| CD8 | converted_iqr | 11 | -2.367 | 27.697 | 0.9338 |
| CD8 | total_n_z | 11 | 0.942 | 11.339 | 0.9356 |
| CD8 | adverse_rate_z | 11 | 12.768 | 10.535 | 0.2564 |
| CD8 | elderly_cohort | 11 | 10.540 | 26.907 | 0.7044 |
| CD8 | explicit_within_24h | 11 | 38.239 | 21.813 | 0.1135 |
| CD3 | language_english | 10 | -56.689 | 85.188 | 0.5245 |
| CD3 | mortality_28_30 | 10 | 116.408 | 75.616 | 0.1623 |
| CD3 | converted_iqr | 10 | -17.318 | 80.901 | 0.8359 |
| CD3 | total_n_z | 10 | 42.692 | 36.416 | 0.2748 |
| CD3 | adverse_rate_z | 10 | 43.456 | 36.611 | 0.2693 |
| CD3 | elderly_cohort | 10 | 17.157 | 84.004 | 0.8433 |
| CD3 | explicit_within_24h | 10 | 88.029 | 74.916 | 0.2738 |
| ALC | language_english | 10 | -126.688 | 108.562 | 0.2768 |
| ALC | mortality_28_30 | 10 | 130.392 | 110.224 | 0.2708 |
| ALC | converted_iqr | 10 | -55.486 | 140.573 | 0.7034 |
| ALC | total_n_z | 10 | 74.126 | 46.753 | 0.1515 |
| ALC | adverse_rate_z | 10 | 70.630 | 51.633 | 0.2085 |
| ALC | elderly_cohort | 10 | 66.963 | 128.044 | 0.6152 |
| ALC | explicit_within_24h | 10 | 129.044 | 102.604 | 0.244 |

## **Supplementary Table S8. Post hoc descriptive hierarchical concordance**

| **study** | **year** | **reported_compartments** | **negative_compartments** | **all_reported_negative** | **at_least_three_lower** | **directions** |
| --- | --- | --- | --- | --- | --- | --- |
| Polilli et al. | 2021 | 4 | 4 | Yes | Yes | ALC=lower; CD3=lower; CD4=lower; CD8=lower |
| Li et al. (Aging) | 2024 | 4 | 3 | No | Yes | ALC=higher; CD3=lower; CD4=lower; CD8=lower |
| Huang et al. | 2024 | 3 | 3 | Yes | Yes | ALC=lower; CD4=lower; CD8=lower |
| Suri et al. | 2024 | 4 | 4 | Yes | Yes | ALC=lower; CD3=lower; CD4=lower; CD8=lower |
| Chen et al. | 2022 | 4 | 4 | Yes | Yes | ALC=lower; CD3=lower; CD4=lower; CD8=lower |
| Li et al. (Front Immunol) | 2025 | 3 | 3 | Yes | Yes | CD3=lower; CD4=lower; CD8=lower |
| Cheng et al. | 2025 | 3 | 3 | Yes | Yes | ALC=lower; CD3=lower; CD4=lower |
| Jia et al. | 2024 | 4 | 4 | Yes | Yes | ALC=lower; CD3=lower; CD4=lower; CD8=lower |
| Dong et al. | 2025 | 4 | 4 | Yes | Yes | ALC=lower; CD3=lower; CD4=lower; CD8=lower |
| Yang et al. | 2023 | 4 | 4 | Yes | Yes | ALC=lower; CD3=lower; CD4=lower; CD8=lower |
| Wei et al. | 2021 | 4 | 4 | Yes | Yes | ALC=lower; CD3=lower; CD4=lower; CD8=lower |

This post hoc analysis was not specified in the PROSPERO protocol. Because ALC, CD3, CD4, and CD8 represent correlated cell compartments, the concordance results were interpreted descriptively and were not treated as an independent line of evidence.

## **Supplementary Table S9. Yang 2023 dispersion sensitivity**

| **Scenario** | **Marker** | **k** | **MD** | **95% CI** | **I² (%)** |
| --- | --- | --- | --- | --- | --- |
| Primary: printed values interpreted as SE and converted to SD | CD4 | 13 | -86.06 | -127.29 to -44.83 | 77.0 |
| Primary | CD8 | 11 | -41.83 | -68.37 to -15.29 | 61.9 |
| Primary | CD3 | 10 | -133.99 | -220.41 to -47.56 | 88.3 |
| Primary | ALC | 10 | -201.46 | -323.04 to -79.88 | 71.7 |
| Yang printed values used as SD | CD4 | 13 | -86.72 | -127.48 to -45.95 | 84.2 |
| Yang printed values used as SD | CD8 | 11 | -41.44 | -66.08 to -16.80 | 62.0 |
| Yang printed values used as SD | CD3 | 10 | -134.62 | -218.31 to -50.92 | 97.8 |
| Yang printed values used as SD | ALC | 10 | -201.98 | -318.42 to -85.53 | 78.8 |
| Yang 2023 excluded | CD4 | 12 | -84.01 | -129.16 to -38.86 | 78.2 |
| Yang 2023 excluded | CD8 | 10 | -42.39 | -72.14 to -12.65 | 65.7 |
| Yang 2023 excluded | CD3 | 9 | -132.42 | -229.90 to -34.95 | 88.8 |
| Yang 2023 excluded | ALC | 9 | -200.63 | -338.73 to -62.54 | 74.0 |

## **Supplementary Table S10. Potential cohort-overlap sensitivity**

| **Scenario** | **Marker** | **k** | **MD** | **95% CI** | **P** | **I² (%)** |
| --- | --- | --- | --- | --- | --- | --- |
| Primary | CD4 | 13 | -86.06 | -127.29 to -44.83 | <0.001 | 77.0 |
| Primary | CD3 | 10 | -133.99 | -220.41 to -47.56 | 0.0066 | 88.3 |
| Preferred overlap sensitivity | CD4 | 12 | -77.48 | -116.25 to -38.70 | 0.0011 | 75.1 |
| Preferred overlap sensitivity | CD3 | 9 | -117.60 | -204.58 to -30.62 | 0.0143 | 87.7 |
| Alternative overlap sensitivity: exclude Li Front | CD4 | 12 | -90.75 | -135.63 to -45.86 | <0.001 | 77.9 |
| Alternative overlap sensitivity: exclude Li Front | CD3 | 9 | -143.69 | -242.69 to -44.69 | 0.0101 | 89.0 |
| Stress test: exclude both Li Front and Cheng | CD4 | 11 | -81.44 | -124.43 to -38.44 | 0.0018 | 76.3 |
| Stress test: exclude both Li Front and Cheng | CD3 | 8 | -125.88 | -228.20 to -23.55 | 0.0227 | 88.4 |

Overlap evidence: Li (Front Immunol) 2025 and Cheng 2025 shared Peking Union Medical College Hospital, ChiCTR2300074175, six authors, and overlapping recruitment periods. Patient-level duplication could not be confirmed or quantified from published reports.

## **Supplementary Table S11. Leave-one-out summary**

| **Marker** | **Number of fits** | **Pooled MD range** | **Overall CI envelope** | **Fits with CI excluding zero** |
| --- | --- | --- | --- | --- |
| CD4 | 13 | -92.68 to -68.95 | -136.18 to -35.23 | All |
| CD8 | 11 | -47.11 to -29.28 | -75.48 to -10.38 | All |
| CD3 | 10 | -153.31 to -101.82 | -243.77 to -27.06 | All |
| ALC | 10 | -231.11 to -160.19 | -358.03 to -51.40 | All |

## **Supplementary Table S12. Leading Baujat heterogeneity/influence contributors**

| **Marker** | **Study** | **Squared Pearson residual** | **Influence on pooled result** |
| --- | --- | --- | --- |
| CD4 | Polilli et al. 2021 | 3.869 | 1.248 |
| CD4 | Cheng et al. 2025 | 3.358 | 0.251 |
| CD4 | Li et al. (Aging) 2024 | 1.156 | 0.089 |
| CD8 | Polilli et al. 2021 | 5.878 | 4.764 |
| CD8 | Li et al. (Aging) 2024 | 1.267 | 0.208 |
| CD8 | Jia et al. 2024 | 0.602 | 0.024 |
| CD3 | Polilli et al. 2021 | 2.724 | 0.985 |
| CD3 | Li et al. (Aging) 2024 | 1.620 | 0.267 |
| CD3 | Cheng et al. 2025 | 1.676 | 0.202 |
| ALC | Polilli et al. 2021 | 2.865 | 0.843 |
| ALC | Li et al. (Aging) 2024 | 1.957 | 0.349 |
| ALC | Dong et al. 2025 | 0.982 | 0.177 |

## **Supplementary Table S13. QUIPS domain-level judgments**

| **Study** | **Participation** | **Attrition** | **Prognostic-factor measurement** | **Outcome measurement** | **Confounding** | **Analysis/reporting** | **Overall descriptive judgment** |
| --- | --- | --- | --- | --- | --- | --- | --- |
| Polilli 2021 | Moderate | Low | Moderate | Low | Moderate | Moderate | Moderate |
| Wei 2021 | Low | Low | Moderate | Low | High | Moderate | High |
| Chen 2022 | Moderate | Low | Moderate | Low | High | Moderate | High |
| Yang 2023 | Moderate | Low | Moderate | Low | High | High | High |
| Suri 2024 | Low | Low | Moderate | Low | Moderate | Moderate | Moderate |
| Huang 2024 | Low | Low | Low | Low | Moderate | Moderate | Moderate |
| Li (Aging) 2024 | Moderate | Low | Moderate | Low | High | High | High |
| Jia 2024 | Moderate | Low | Low | Low | High | Moderate | High |
| Miao 2024 | Moderate | Low | Moderate | Low | Moderate | Moderate | Moderate |
| Dong 2025 | Moderate | Low | High | Low | High | Moderate | High |
| Du 2025 | Moderate | Low | Moderate | Low | Moderate | Moderate | Moderate |
| Li (Front Immunol) 2025 | Moderate | Low | Moderate | Moderate | Moderate | High | High |
| Cheng 2025 | Low | Low | Moderate | Moderate | High | High | High |

## Supplementary Table S14. Marker-specific evidence robustness and limitations

Formal GRADE categories were not assigned because the principal pooled estimates are unadjusted survivor–non-survivor contrasts and the included studies do not provide a uniform phase of prognostic-factor investigation with comparable multivariable adjustment. The table therefore summarizes marker-specific robustness and limitations using QUIPS, prediction intervals, sensitivity analyses, adjusted evidence, and other prespecified or post hoc diagnostics.

| **Marker** | **Main MD (95% CI)** | **95% PI** | **High descriptive QUIPS risk** | **No-conversion sensitivity** | **Lower-risk sensitivity** | **Adjusted evidence** | **Additional concerns** | **Descriptive interpretation** |
| --- | --- | --- | --- | --- | --- | --- | --- | --- |
| CD4 | -86.06 (-127.29 to -44.83) | -218.44 to 46.31 | 8/13 studies | k=5; -60.17 (-92.34 to -28.01) | k=5; -106.57 (-195.02 to -18.12) | Mixed; selected studies positive, largest multicenter cohort null for CD4/total T-cell after adjustment | 11/13 cohorts from China; PI crosses zero | Most consistent association across tested restrictions |
| CD8 | -41.83 (-68.37 to -15.29) | -114.26 to 30.60 | 7/11 studies | k=3; -44.96 (-95.88 to 5.97) | k=4; -60.97 (-145.90 to 23.95) | Inconsistent; no uniform independent effect | 11/13-study evidence base geographically concentrated; PI crosses zero | Supportive but less stable after conversion and QUIPS restrictions |
| CD3 | -133.99 (-220.41 to -47.56) | -390.53 to 122.56 | 8/10 studies | k=4; -113.76 (-217.70 to -9.83) | k=2; -183.22 (-1988.34 to 1621.90) | Inconsistent; total T-cell effect not retained in largest cohort | I² 88.3%; Yang dispersion ambiguity; possible small-study effects; PI crosses zero | Substantial uncertainty despite negative pooled association |
| ALC | -201.46 (-323.04 to -79.88) | -539.77 to 136.85 | 7/10 studies | k=2; -179.29 (-1178.94 to 820.36) | k=3; -322.14 (-828.60 to 184.32) | Limited and inconsistent adjusted evidence | Conversion dependence; possible small-study effects; PI crosses zero | Most sensitive to sparse and converted evidence |

## Supplementary Table S15. Source data and analysis code inventory

| **Path** | **File** | **Purpose / corresponding output** |
| --- | --- | --- |
| / | README.txt | Archive scope, execution instructions, and output locations. |
| / | Install Packages.R | Installs required R packages; ragg and svglite remain optional. |
| / | Run All Analyses.R | Master script; sequentially reproduces the primary analyses, key sensitivity analyses, and statistical figures. |
| /R | 00 Setup.R | Loads packages, data, reference values, and global settings. |
| /R | 01 Functions.R | Core REML modified Hartung-Knapp, ratio-of-means, Egger, and helper functions. |
| /R | 09 Formal Analysis v4 1.R | Primary meta-analyses, restricted analyses, Yang and overlap sensitivities, leave-one-out, RoM, Egger, meta-regression, and Baujat outputs. |
| /R | 11 QUIPS High Risk Sensitivity.R | Derives the high-risk exclusion set directly from QUIPS judgments and refits CD4, CD8, CD3, and ALC; supplies Supplementary Figure S9 data. |
| /R | 10 Figures v4 1.R | Generates Figures 2-4 and Supplementary Figures S1-S9 from the analysis objects. |
| /data | analysis data.csv | Harmonized study-marker data with means, SDs, sample sizes, effect inputs, and audit fields. |
| /data | analysis data v4 0.csv | Analysis-ready dataset with sampling, language, mortality-window, conversion, and special-population indicators. |
| /data | raw extraction.csv | Original reported group statistics, source locations, and extraction metadata. |
| /data | data dictionary.csv | Variable definitions for the analytical datasets. |
| /data | reference main results.csv | Four locked primary pooled results used for automated reproduction checks. |
| /data | QUIPS domain judgments.csv | Domain-level and descriptive overall QUIPS judgments used by the risk-of-bias analysis. |
| /data | Li Cheng overlap evidence.csv | Audit information supporting the potential cohort-overlap sensitivity scenarios. |

# **Supplementary figure legends**

Supplementary Figure S1. Funnel plots for (A) CD4, (B) CD8, (C) CD3, and (D) ALC. Asymmetry is descriptive; Egger tests suggested possible small-study effects for CD3 and ALC only.

Supplementary Figure S2. Leave-one-out analyses for (A) CD4, (B) CD8, (C) CD3, and (D) ALC. The solid vertical line indicates MD = 0 and the dashed vertical line indicates the overall pooled MD.

Supplementary Figure S3. Language-stratified pooled estimates for English- and Chinese-language studies. Publication language did not correspond directly to geographic setting because most English-language studies were conducted in China.

Supplementary Figure S4. Sensitivity analysis restricted to studies with explicit sampling within 24 h or on day 1.

Supplementary Figure S5. Yang 2023 dispersion sensitivity comparing the primary SE-to-SD interpretation, direct use of the printed values as SD, and exclusion of Yang 2023.

Supplementary Figure S6. Potential Li–Cheng cohort-overlap sensitivity for (A) CD4 and (B) CD3 under mutually exclusive cohort-removal scenarios.

Supplementary Figure S7. Baujat diagnostics for (A) CD4, (B) CD8, (C) CD3, and (D) ALC.

Supplementary Figure S8. QUIPS domain-level risk-of-bias judgments for the 13 included studies. L, M, and H denote low, moderate, and high risk, respectively.

Supplementary Figure S9. Sensitivity analysis excluding studies with a high overall descriptive QUIPS judgment. CD4 remained significantly lower in non-survivors; estimates for CD8, CD3, and ALC remained negative but were imprecise because only four, two, and three studies remained, respectively.
